# Family Caregivers of People with Dementia Have Poor Sleep Quality: A Nationwide Population-Based Study

**DOI:** 10.3390/ijerph182413079

**Published:** 2021-12-11

**Authors:** Min Ji Song, Ji Hyun Kim

**Affiliations:** 1 Department of Neurology, Korea University Anam Hospital, Korea University College of Medicine, Seoul 02841, Korea; songminji1034@gmail.com; 2 Department of Neurology, Korea University Guro Hospital, Korea University College of Medicine, Seoul 08308, Korea

**Keywords:** people with dementia, cohabitating family caregivers, noncohabitating family caregivers, sleep quality

## Abstract

Previous studies have documented cognitive impairments, psychological stress, and depressive symptoms in family caregivers of people with dementia (PWD), which could be attributed to their sleep disturbances. Notwithstanding the increasing recognition of poor sleep quality and sleep disturbances in family caregivers of PWD, their association has not been tested yet using population-representative samples. We conducted a retrospective, cross-sectional study using population-based data from the 2018 Korean Community Health Survey. Sociodemographic, mental health-related, and physical health-related variables as well as sleep quality evaluated by the Pittsburgh Sleep Quality Index (PSQI) were compared between 2537 cohabitating caregivers of PWD, 8864 noncohabitating caregivers of PWD, and 190,278 non-caregivers. Two sets of multivariable logistic regressions were conducted to examine the associations between dementia caregiving and poor sleep quality in cohabitating and noncohabitating caregivers versus noncaregivers. Both cohabitating and noncohabitating caregivers had higher global PSQI scores and higher prevalence of poor sleep quality (PSQI score > 5) than did noncaregivers. Multivariable logistic regressions adjusted for potential confounders revealed that cohabitating caregivers (odds ratio (OR) 1.26, 95% confidence interval (CI) 1.15–1.38) and noncohabitating caregivers (OR 1.15, CI 1.10–1.21) were significantly associated with poor sleep quality. Our results showed that both cohabitating and noncohabitating caregivers of PWD experienced overall poorer sleep quality compared to noncaregivers, indicating the deleterious effect of dementia caregiving on sleep quality, regardless of living arrangements. Given the high prevalence of poor sleep quality in family caregivers of PWD and the increasing awareness of the serious health consequences of poor-quality sleep, physicians should consider active sleep interventions to promote health and wellbeing not only for the dementia patients but also for family caregivers.

## 1. Introduction

Dementia affects approximately 47 million adults worldwide as of 2016 [1]. The estimated number of people with dementia (PWD) in 2015 is approximately 0.6 million in South Korea, with this number expected to increase by 2030 to 1.3 million [1]. Dementia poses a growing public health concern and is one of the leading causes of morbidity and mortality [2]. In 2013, the annual cost for dementia in South Korea was estimated to be about USD 10 billion, which represented approximately 0.7% of its gross domestic product [1]. Measurement of the disease burden by calculating disability-adjusted life years indicated that dementia is a very burdensome disease not only to PWD but also to their family members and informal caregivers [2].

PWD eventually become physically dependent on caregivers and exhibit emotional and behavioral symptoms such as depression, sleep disturbance, physical or verbal aggression, and resistance to care [3]. Informal caregiving of PWD frequently occurs over years to decades and is primarily taken charge of by their family members such as spouses, siblings, children, and children-in-law. Caregiving of PWD is complicated, unpredictable, and emotionally, physically, and financially demanding. A meta-analytic study indicated that caregivers’ overall health is generally poor, which was closely associated with their depressive symptoms, care recipients’ behavioral problems, and dementia-related stressors [4]. High or very high levels of emotional and physical stresses were reported in 59% and 38% of family caregivers of PWD, respectively [2].

In the United States, the loss of independent activities of daily living in PWD has prompted 16.3 million adults to become informal caregivers to their parent, spouse, or friend who has Alzheimer disease or other dementias [2]. In 2019, the Alzheimer’s Association estimated that family and other unpaid caregivers on average spent 21.9 h per week or 1139 h per year caring for PWD [2]. Family caregivers of PWD are more likely than family caregivers of people without dementia to help with emotional or mental health problems (41% versus 16%) and behavioral issues (15% versus 4%) [2]. Previous studies also documented psychological stress, depressive symptoms, and cognitive impairments in verbal memory, attention, and executive function in family caregivers of PWD [5,6,7,8,9,10,11,12], which may be attributed to their sleep disturbances [13,14,15,16,17,18,19,20].

Notwithstanding the increasing recognition of poor sleep quality and sleep disturbances in family caregivers of PWD, their association has not been tested yet using population-representative samples. We aimed to replicate the previous finding of poor sleep quality in family caregivers of PWD using a population-based, large-sample dataset. Given that noncohabitating family caregivers were more likely to experience worry, stress, and depression than compared to cohabitating caregivers [21], we hypothesized that not only cohabitating caregivers but also noncohabitating caregivers have poorer sleep quality compared to noncaregivers [22].

## 2. Materials and Methods

### 2.1. Study Population

For this study, we acquired data from the 2018 Korean Community Health Survey (KCHS), a nationwide cross-sectional survey carried out by the Korea Centers for Disease Control and Prevention. This community-based health interview survey is conducted annually since 2008 by 254 community health centers from the 17 metropolitan cities and provinces, 35 community colleges, and 1500 interviewers in order to explore the patterns of personal lifestyle, mental and physical health-related behaviors, and disease prevalence and morbidity in adults aged ≥19 years [23]. The sample size for each of the 254 community units is 900 participants, and the expected number of respondents in this survey is 228,600. KCHS employed a two-stage sampling process to ensure that the sample units are representative of the general population [24]. The first stage involves selection of a sample area (tong/ban/ri) as a primary sample unit according to the number of households using a probability proportional to size sampling technique. The second stage of sampling process includes selection of sample households in each sample area using systematic sampling methods. In order to ensure the samples to be statistically representative of the general population, survey data were weighted by reference to sampling design [24].

Exclusion criteria were the following: (1) uninhabitable areas owing to redevelopment or reconstruction; (2) households in the nonresidential areas (e.g., business district and industrial complex); (3) residences for specific groups (e.g., lepers colony, dormitory, and religious communities); and (4) households where the interviewer could not contact the family members after visiting the household more than three times. Data were collected by technicians trained in conducting computer-assisted in-person interviews. Among a total of 228,340 participants in the 2018 KCHS, 26,661 were excluded because they did not fill out the questionnaire variables listed in Table 1 and Table 2. Accordingly, 201,679 participants without missing variables were finally selected for statistical analysis.

KCHS data are publicly available, and all data are completely anonymized before its release. This study was exempted from the need for an Institutional Review Board review since it did not correspond to research on human participants, according to Section 2.2 of the Enforcement Rule of Bioethics and Safety Act in Korea. All procedures of the survey were conducted in accordance with the ethical standards of the National Research Committee and the 1964 Helsinki Declaration and its later amendments.

### 2.2. Independent Variable: Dementia Caregiving

Participants were asked if there is a patient who had been diagnosed with dementia by a physician and requires caregiving in their family (parents, spouse, siblings, and children). The responses were either yes or no. If participants answered yes, they were further inquired about living arrangements of the family caregivers. The responses were either living with the PWD (cohabitating caregivers) or living apart from the PWD (noncohabitating caregivers). If participants answered no, they were categorized as noncaregivers.

### 2.3. Dependent Variable: Sleep Quality

Sleep quality was assessed using the Korean version of the Pittsburgh Sleep Quality Index (PSQI), which was validated with high specificity and sensitivity [25]. The PSQI is the most extensively used self-administered questionnaire for assessment of sleep quality and patterns during the past month [26]. It provides a reliable and standardized measure for discriminating good sleepers from poor sleepers and has high internal consistency and strong test-retest reliability and validity [27]. Specifically, PSQI comprises 19 items regarding seven sleep components: subjective sleep quality, sleep latency, sleep duration, sleep efficiency, sleep disturbances, use of sleep medication, and daytime dysfunction [26]. Each component was ranked on a 4-point scale ranging from 0 to 3. A global PSQI score for the assessment of overall sleep quality can be determined by adding all seven component scores, resulting in a final score ranging from 0 to 21. A global PSQI score of 5 or below generally indicates good sleep, and a score of 6 or above indicates poor sleep [28].

### 2.4. Covariates

Sociodemographic variables included sex, age, body mass index, residence area (urban or rural), education level (elementary school or lower, middle school, high school, or college or higher), marital status (living with spouse or living alone), employment status (employed or unemployed), and household income (low, middle-low, middle-high, or high).

Physical health-related variables included risky drinking (12 or more drinking episodes in which five or more alcoholic glasses were consumed during the last year) [29], smoking status (current smoker or non-smoker/ex-smoker), regular exercise (at least 30 min of walking for at least 5 days per week) [30], and presence of hypertension and diabetes mellitus.

Mental health-related variables included perceived stress, perceived health status, subjective cognitive decline, and depression. Perceived stress was assessed by asking the following question: “How stressed do you feel in your daily life?” The responses were categorized as yes (severe or very severe) or no (rare or mild). Perceived health status was assessed by asking the following question, “How do you rate your health status in general?” The responses were categorized as good (very good or good), moderate, or bad (bad or very bad). According to the cognitive decline module of the Behavioral Risk Factor Surveillance System [31], subjective cognitive decline was determined by asking the single question, “Have you experienced frequent or worsening of memory loss or confusion during the last year?” The responses were either yes or no. Depressive symptoms were evaluated by using the Patient Health Questionnaire-9 (PHQ-9), a widely adopted scale in population-based studies [32]. The Korean version of the PHQ-9 used in this survey was validated previously [33]. Nine items were measured to evaluate depressive symptoms during the past two weeks and were scored on a scale ranging from 0 to 3 (0 = none; 1 = several days; 2 = more than 7 days; and 3 = nearly every day). A global PHQ-9 score of ≥10 indicates the presence of depression.

### 2.5. Statistics

Descriptive statistics illustrated the baseline characteristics of the study population. Sociodemographic, physical health-related, and mental health-related variables as well as sleep quality measures (PSQI scores) were compared between cohabitating caregivers, noncohabitating caregivers, and noncaregivers by using one-way analysis of variance with Bonferroni correction, chi-square test, or Fisher’s exact test where appropriate.

Two sets of multivariable logistic regressions were conducted to examine the associations between dementia caregiving and overall sleep quality (i.e., good sleep vs. poor sleep) in cohabitating and noncohabitating caregivers versus noncaregivers. Model 1 was adjusted for sex, age, and body mass index. Model 2 was adjusted for Model 1 variables and socioeconomic variables (residence area, education level, marital status, employment status, and household income). Model 3 was adjusted for Model 2 variables, physical health-related variables (risky drinking, smoking, regular exercise, hypertension, and diabetes mellitus), and mental health-related variables (perceived status of health, perceived level of stress, subjective cognitive decline, and depression). Results were expressed as adjusted odds ratio (OR) and 95% confidence interval (CI). A *p* < 0.05 indicates statistical significance in all tests. Statistical analyses were conducted using the Statistical Package for Social Sciences (version 26.0; IBM, Armonk, NY, USA).

## 3. Results

The sociodemographic, physical health-related, and mental health-related variables as well as the PSQI score of the cohabitating caregivers of PWD (*n* = 2537), noncohabitating caregivers of PWD (*n* = 8864), and noncaregivers (*n* = 190,278) are presented in Table 1. Three groups differed in age, residence area, education level, marital status, employment status, household income level, and health-related behaviors (risky drinker and regular exercise) (all *p* < 0.001). Hypertension and diabetes mellitus were more frequently observed in cohabitating caregivers than noncohabitating caregivers and noncaregivers (all *p* < 0.001). Noncaregivers had higher prevalence of hypertension (*p* < 0.001) and diabetes mellitus (*p* = 0.001) than compared to noncohabitating caregivers. These results may be due to significant difference in age between noncaregivers (53.2 ± 17.1) and noncohabitating caregivers (50.5 ± 14.8, *p* < 0.001). Cohabitating caregivers were found to have higher perceived stress, poorer perceived health status, and higher prevalence of subjective cognitive decline and depression than compared to noncohabitating caregivers and noncaregivers (all *p* < 0.001). Noncohabitating caregivers had higher perceived stress (*p* < 0.001) and higher prevalence of subjective cognitive decline (*p* < 0.001) than did noncaregivers. There was no difference in the prevalence of depression between noncohabitating caregivers and noncaregivers (*p* > 0.05).

Comparisons of the global PSQI score and component scores between the groups are summarized in Table 2. The global PSQI score was higher in cohabitating caregivers (5.6 ± 3.3) than in noncohabitating caregivers (4.9 ± 2.9, *p* < 0.001) and noncaregivers (4.7 ± 2.9, *p* < 0.001). The global PSQI score was higher in noncohabitating caregivers than in noncaregivers (*p* < 0.001). As expected, poor sleeper (PSQI score > 5) was more prevalent in cohabitating caregivers (41.7%) than in noncohabitating caregivers (31.9%, *p* < 0.001) and noncaregivers (29.8%, *p* < 0.001). Noncohabitating caregivers had higher prevalence of poor sleeper than did noncaregivers (*p* < 0.001). The scores for seven PSQI components were higher in cohabitating caregivers compared with noncaregivers (all *p* < 0.001), indicating that cohabitating caregivers had poorer overall sleep quality than did noncaregivers. Noncohabitating caregivers had higher scores in sleep duration, sleep disturbance, and daytime dysfunction than did noncaregivers (all *p* < 0.001).

Table 3 shows significant associations between poor sleep quality measured by the PSQI and family caregivers of PWD after adjustment for sex, age, and body mass index (Model 1); Model 1 variables and socioeconomic variables (Model 2); and Model 2 variables and physical and mental health-related variables (Model 3). Cohabitating caregivers had higher prevalence of poor sleep quality (PSQI score > 5) than compared to noncaregivers (Model 1: OR 1.58, CI 1.45–1.71; Model 2: OR 1.55, CI 1.43–1.69; Model 3: OR 1.25, CI 1.15–1.37). Likewise, noncohabitating caregivers had a higher prevalence of poor sleep quality than compared to noncaregivers (Model 1: OR 1.15, CI 1.10–1.20; Model 2: OR 1.22, CI 1.16–1.28; Model 3: OR 1.15, CI 1.10–1.21).

## 4. Discussion

To our knowledge, this study is the first to demonstrate the associations between poor sleep quality and family caregivers of PWD using population-based samples. The main finding is that cohabitating caregivers of PWD experienced overall poor sleep quality relative to noncaregivers even after adjustment for potential confounders. Moreover, poor sleep quality was more frequently observed in noncohabitating caregivers of PWD than in noncaregivers, suggesting that having a dementia patient in the family could result in poorer sleep quality, irrespective of living arrangements.

The detrimental influences of informal caregiving on the physical, psychological, and social wellbeing of dementia caregivers are well documented in previous investigations. Specifically, family caregivers of PWD were found to have higher risks of atherosclerosis [34,35,36,37], poorer physical health conditions [4,10,38], cognitive impairments [6,7,8,9], and poorer psychological health including stress [39], depression, and anxiety [13,40,41,42]. Consistent with the aforementioned studies, our study showed that caregivers of PWD had higher prevalence of hypertension, diabetes mellitus, subjective cognitive decline, and depression than noncaregivers. In addition, caregivers of PWD experienced higher stress level and poorer health status compared to noncaregivers, further corroborating that dementia caregiving is associated with cognitive impairment and poor physical and psychological health statuses.

It has been repeatedly shown in prior case-control studies using a small number of participants that family caregivers of PWD reported less total sleep time compared to noncaregivers, as measured by self-report [43,44], actigraphy [44], and polysomnography [45,46,47]. A recent meta-analysis showed that family caregivers of PWD had shorter sleep duration akin to losing 2.42 to 3.50 h per a week relative to age-matched noncaregivers [18]. However, this association was not replicated in other studies [34,48,49]. The incongruent results between the studies might be partly ascribed to the use of small samples and differences in confounders affecting sleep duration and quality included in the statistics, such as socioeconomic variables, comorbid psychiatric and medical disorders, and the use of psychotropic and hypnotic medications. Our study using population-representative samples found that cohabitating caregivers had higher PSQI subscores of the sleep duration, sleep latency, and sleep efficiency than compared to noncaregivers, pointing to a higher prevalence of insufficient sleep in cohabitating caregivers of PWD.

Sleep quality was investigated in family caregivers of PWD in recent studies using PSQI. The studies have consistently shown that family caregivers of PWD had higher PSQI global score than noncaregivers, implying that family caregivers of PWD have overall poor sleep quality [12,45,46,49,50,51,52,53,54]. As expected, the pooled analysis in a meta-analytic study confirmed that 1686 dementia caregivers reported significantly worse global PSQI scores (5.58 ± 3.30) relative to 478 noncaregivers (4.79 ± 3.17) [18]. Moreover, all seven component scores of the PSQI were significantly higher in caregivers than those of noncaregivers, which is in good accordance with our study. In our study, multivariable logistic regression controlling for potential confounders, including sociodemographic, physical health-related, and mental health-related variables, yielded strong support for the associations between caregivers of PWD and poor sleep quality. Taken together, our results suggest that cohabitating caregivers of PWD suffer from not only insufficient sleep and poor sleep efficiency but also increased sleep disturbances and daytime dysfunctions. Given that caregivers who underwent behavioral interventions were found to have better sleep quality than those who did not receive a sleep intervention [55,56,57,58,59], family caregivers of PWD need to be more precisely evaluated for poor sleep quality and targeted for active sleep interventions to improve their sleep quality and health [18].

The majority of dementia caregiving is provided by unpaid caregivers, most of whom are family members caring for PWD at home [2]. Living arrangements of the family caregivers consist of caregivers living at home with the PWD, those living in close proximity accessible for supportive and direct care on a daily basis, and those not having easy access to the PWD [60]. The main cause of poor sleep quality observed in cohabitating caregivers may be disrupted sleep due to nighttime behavioral and psychological problems by the PWD [17,61,62]. Interestingly, a few studies have shown that noncohabitating caregivers of PWD experienced the same levels of depression and stress as well as poor sleep quality, as did cohabitating caregivers [22,62]. In parallel with the abovementioned studies, our study revealed that noncohabitating caregivers had higher perceived stress and higher prevalence of subjective cognitive decline and poor sleep quality than did noncaregivers. Furthermore, noncohabitating caregivers reported higher scores in three PSQI components including sleep duration, sleep disturbance, and daytime dysfunction than compared to noncaregivers. The fully adjusted logistic regression model confirmed the association between poor sleep quality and noncohabitating caregivers. High perceived stress, increased depressive symptoms, and badly self-reported health may account for poor sleep quality observed in noncohabitating caregivers of PWD [21,22,63]. Given the fundamental role of good sleep quality for maintaining health, our findings exaggerate the importance of evaluating caregivers living apart from the PWD for sleep quality and problems with the same level of concern as one would have for those living with the PWD.

The strengths of this study may be the use of a population-based, large-sample dataset and the adoption of a sampling process representative of the general population. Furthermore, this survey offered information about a number of confounders related with sleep quality, which allowed us to explore the association between poor sleep quality and dementia caregiving using multiple statistical adjustments. Our results need to be interpreted within the confines of at least five potential limitations. First, the cross-sectional design of the study does not enable us to make causal inferences with regard to the directionality of associations. In addition, given the differences in ethnicity and social environment for dementia caregiving, further investigations using other populations should be required to confirm our results. Second, due to the fact that data were collected via self-administered questionnaires, recall bias resulting in the probability of under-reporting or over-reporting cannot be entirely discounted. In addition, the lack of objective measures of sleep quality does not allow us to verify the accuracy of self-reported data. The PSQI is a subjective measure of sleep quality and may reflect the overall psychological state of the person, rather than actual quality or quantity of sleep. Subjective measures for sleep (i.e., PSQI scores) have shown weak or inconsistent correlations with objective measures (e.g., actigraphy and polysomnography) [64,65,66]. Therefore, further studies adopting both types of sleep measures are warranted to confirm our findings. Third, although the PSQI cutoff score of 5 is the most widely used in sleep medicine, there has been some debate as to whether this cutoff score could be an optimal threshold indicating good or poor sleep quality. Some authors suggested a higher threshold to substantially differentiate good sleepers from poor sleepers [67,68]. Moreover, a recent study found a discrepancy between the PSQI scores on work-free days and workdays, suggesting the influences of chronotype and social jetlag on sleep quality [69]. Fourth, a dichotomous question for living with or apart from the PWD limits comprehensive assessment of caregivers’ burden. Moreover, sleep quality measured during the past one month might be insufficient for reflecting sleep quality of caregiving that commonly occurs over many years. Thus, our findings from a cross-sectional survey need confirmation with future studies incorporating a longitudinal design and additional information regarding caregivers’ burden, such as caregiving burden scale, time spent in caregiving, quality of life, and detailed cognitive and psychological measures. Finally, this survey did not collect more specific information with regard to maintenance of sleep and development of sleep problems, such as sleep apnea, insomnia, nocturia, and the use of analgesics and psychotropic drugs (antipsychotics, anxiolytics, and antidepressants), which could compromise the results.

## 5. Conclusions

Our findings from a nationwide population-based study using the PSQI substantiate the previous finding that family caregivers of PWD experience overall poorer sleep quality compared to noncaregivers. Specifically, these dementia caregivers more frequently reported not only insufficient sleep and poor sleep efficiency but also increased sleep disturbances and daytime dysfunctions compared with noncaregivers. It is also of note that family caregivers living apart from the PWD were found to experience the same level of poor sleep quality as caregivers living with the PWD, indicating the deleterious effect of dementia caregiving on sleep quality regardless of living arrangements. For proper treatment and management of family caregivers’ sleep problems, it is the first step to comprehensively evaluate the nature and severity of their sleep disturbances and related burdens. Given the high prevalence of poor sleep quality in family caregivers of PWD and the increasing awareness of serious health consequences of poor quality sleep [19,70], physicians should accurately assess the health aspects of family caregivers and consider active sleep interventions to promote health and wellbeing not only for the dementia patients but also for the family caregivers [18]. Further studies with a longitudinal study design, theoretical framework, objective sleep measures, and important covariates such as preexisting sleep disorders and prescribed drugs are needed to strengthen the knowledge and evidence regarding sleep disturbances in family caregivers of PWD.

## Figures and Tables

**Table 1:** Baseline characteristics of the cohabitating caregivers, noncohabitating caregivers, and noncaregivers.

Variables	Cohabitating Caregivers (*n* = 2537)	Noncohabitating Caregivers (*n* = 8864)	Noncaregivers (*n* = 190,278)	*p*
Age (y)	60.1 ± 17.4	50.5 ± 14.8	53.2 ± 17.1	<0.001
Sex				0.848
Male	1196 (47.1)	4124 (46.5)	88,635 (46.6)	
Female	1341 (52.9)	4740 (53.5)	101,643 (53.4)	
Body mass index (kg/m^2^)	23.6 ± 3.4	23.7 ± 3.3	23.7 ± 3.4	0.158
Residence area				<0.001
Urban	1245 (49.1)	5742 (64.8)	111,828 (58.8)	
Rural	1292 (50.9)	3122 (35.2)	78,450 (41.2)	
Education				<0.001
Elementary school or lower	766 (30.2)	971 (11.0)	39,807 (20.9)	
Middle school	365 (14.4)	942 (10.6)	22,134 (11.6)	
High school	737 (29.1)	3124 (35.2)	56,416 (29.7)	
College or higher	669 (26.3)	3827 (43.2)	71,921 (37.8)	
Marital status				<0.001
Married (living with spouse)	1754 (69.1)	6499 (73.3)	130,311 (68.5)	
Living alone ^a^	783 (30.9)	2365 (26.7)	59,967 (31.5)	
Employment status				<0.001
Employed	1315 (51.8)	5967 (67.3)	120,550 (63.4)	
Unemployed	1222 (48.2)	2897 (32.7)	69,728 (39.6)	
Household income				<0.001
Low	976 (38.5)	1506 (17.0)	50,654 (26.6)	
Middle low	685 (27.0)	2357 (26.6)	54,170 (28.5)	
Middle high	527 (20.7)	2881 (32.5)	52,995 (27.8)	
High	349 (13.8)	2120 (23.9)	32,459 (17.1)	
Smoking				0.173
Current smoker	421 (16.6)	1592 (18.0)	34,305 (18.0)	
Non/ex-smoker	2116 (83.4)	7272 (82.0)	155,973 (82.0)	
Risky drinker				<0.001
Yes	560 (22.1)	2707 (30.5)	52,915 (27.8)	
No	1977 (77.9)	6157 (69.5)	137,363 (72.2)	
Regular exercise				<0.001
Yes	1044 (41.2)	4117 (46.4)	86,320 (45.4)	
No	1493 (58.8)	4747 (53.6)	103,958 (54.6)	
Hypertension				<0.001
Yes	882 (34.8)	1952 (22.0)	51,092 (26.9)	
No	1655 (65.2)	6912 (78.0)	139,186 (73.1)	
Diabetes mellitus				<0.001
Yes	399 (15.7)	848 (9.6)	20,286 (10.7)	
No	2138 (84.3)	8016 (90.4)	169,992 (89.3)	
Subjective cognitive decline				<0.001
Yes	748 (29.5)	1684 (19.0)	33,104 (17.4)	
No	1789 (70.5)	7180 (81.0)	157,174 (82.6)	
Perceived stress				<0.001
Yes	854 (33.7)	2295 (25.9)	44,123 (23.2)	
No	1683 (66.3)	6569 (74.1)	146,155 (76.8)	
Perceived health status				<0.001
Good	712 (28.1)	3197 (36.1)	69,418 (36.5)	
Moderate	1067 (42.0)	4242 (47.8)	85,727 (45.1)	
Bad	758 (29.9)	1425 (16.1)	35,133 (18.4)	
PHQ-9 global score	3.4 ± 4.6	2.3 ± 3.2	2.1 ± 3.1	<0.001
Depression				<0.001
Yes (PHQ9 score ≥ 10)	198 (7.8)	307 (3.5)	6408 (3.4)	
No (PHQ9 score 0–9)	2339 (92.2)	8557 (96.5)	183,870 (96.6)	

Note: Data are presented as number (percent) or mean ± standard deviation. ^a^ Living alone indicates participants who never married or are separated, divorced, or widowed. Group comparisons were performed using one-way analysis of variance, chi-square test, or Fisher’s exact test where appropriate. PHQ-9, Patient Health Questionnaire-9.

**Table 2:** Comparisons of global PSQI and seven component scores among cohabitating caregivers, noncohabitating caregivers, and noncaregivers.

PSQI	Cohabitating Caregivers (*n* = 2537)	Noncohabitating Caregivers (*n* = 8864)	Noncaregivers (*n* = 190,278)	*p*
PSQI global score	5.56 ± 3.30 ^a b^	4.90 ± 2.86 ^c^	4.74 ± 2.88	<0.001
Good sleep (PSQI ≤ 5)	1479 (58.3)	6036 (68.1)	133,577 (70.2)	<0.001
Poor sleep (PSQI > 5)	1058 (41.7)	2828 (31.9)	56,701 (29.8)	
Sleep quality	1.25 ± 0.73 ^a b^	1.11 ± 0.69	1.11 ± 0.68	<0.001
0: very good	298 (11.7)	1374 (15.5)	27,980 (14.7)	
1: fairly good	1461 (57.6)	5471 (61.7)	119,349 (62.7)	
2: fairly bad	629 (24.8)	1716 (19.4)	36,749 (19.3)	
3: very bad	149 (5.9)	303 (3.4)	6200 (3.3)	
Sleep latency (score)	1.05 ± 1.08 ^a b^	0.89 ± 1.01	0.89 ± 1.00	<0.001
0: 0	1032 (40.7)	4125 (46.5)	87,737 (46.1)	
1: 1–2	725 (28.6)	2570 (29.0)	56,589 (29.7)	
2: 3–4	404 (15.9)	1216 (13.7)	25,887 (13.6)	
3: 5–6	376 (14.8)	953 (10.8)	20,065 (10.6)	
Sleep duration (h)	1.24 ± 0.91 ^a^	1.21 ± 0.82 ^c^	1.16 ± 0.82	<0.001
0: > 7	400 (15.8)	1390 (15.7)	33,969 (17.9)	
1: 6–7	1308 (51.5)	5039 (56.8)	108,629 (57.1)	
2: 5–6	456 (18.0)	1584 (17.9)	31,024 (16.3)	
3: < 5	373 (14.7)	851 (9.6)	16,656 (8.7)	
Sleep efficiency (%)	0.15 ± 0.49 ^a b^	0.10 ± 0.40	0.11 ± 0.41	<0.001
0: ≥ 85	2248 (88.6)	8181 (92.3)	175,340 (92.2)	
1: 75–84	213 (8.4)	507 (5.7)	10,932 (5.7)	
2: 65–74	48 (1.9)	113 (1.3)	2494 (1.3)	
3: ≤ 65	28 (1.1)	63 (0.7)	1512 (0.8)	
Sleep disturbance (score)	1.09 ± 0.59 ^a b^	0.98 ± 0.55 ^c^	0.95 ± 0.56	<0.001
0: 0	290 (11.4)	1364 (15.4)	34,028 (17.9)	
1: 1–9	1753 (69.1)	6316 (71.2)	132,462 (69.6)	
2: 10–18	457 (18.0)	1149 (13.0)	22,923 (12.0)	
3: 19–27	37 (1.5)	35 (0.4)	865 (0.5)	
Use of sleeping medication	0.19 ± 0.69 ^a b^	0.08 ± 0.44	0.09 ± 0.47	<0.001
0: not during the past month	2346 (92.5)	8543 (96.3)	182,682 (96.0)	
1: less than once a week	33 (1.3)	95 (1.1)	2030 (1.1)	
2: once or twice a week	30 (1.2)	67 (0.8)	1573 (0.8)	
3: three or more times a week	128 (5.0)	159 (1.8)	3993 (2.1)	
Daytime dysfunction	0.59 ± 0.86 ^a b^	0.53 ± 0.77 ^c^	0.44 ± 0.73	<0.001
0: very good	1571 (61.9)	5529 (62.4)	131,251 (69.0)	
1: fairly good	545 (21.5)	2181 (24.6)	38,489 (20.2)	
2: fairly bad	315 (12.4)	971 (11.0)	17,273 (9.1)	
3: very bad	106 (4.2)	183 (2.1)	3265 (1.7)	

Note: Data are presented as number (percent) or mean ± standard deviation. Group comparisons were made using one-way analysis of variance with Bonferroni correction. ^a^ Corrected *p* < 0.05, cohabitating caregivers versus noncaregivers; ^b^ corrected *p* < 0.05, cohabitating caregivers versus noncohabitating caregivers; ^c^ corrected *p* < 0.05, noncohabitating caregivers versus noncaregivers. PSQI, Pittsburgh Sleep Quality Index.

**Table 3:** Results of multivariable logistic regression showing significant associations between poor sleep quality and family caregivers of people with dementia.

**Cohabitating Caregivers versus Noncaregivers**						
	**Model 1**		**Model 2**		**Model 3**	
	**OR**	**95% CI**	**OR**	**95% CI**	**OR**	**95% CI**
Poor sleep (PSQI > 5)	1.58	1.45–1.71	1.55	1.43–1.69	1.25	1.15–1.37
**Noncohabitating Caregivers versus Noncaregivers**						
	**Model 1**		**Model 2**		**Model 3**	
	**OR**	**95% CI**	**OR**	**95% CI**	**OR**	**95% CI**
Poor sleep (PSQI > 5)	1.15	1.10–1.20	1.22	1.16–1.28	1.15	1.10–1.21

Note: Model 1 was adjusted for sex, age, and body mass index. Model 2 was adjusted for Model 1 variables and socioeconomic variables (residence area, education level, marital status, employment status, and household income). Model 3 was adjusted for Model 2 variables and physical health-related variables (risky drinking, smoking, regular exercise, hypertension, and diabetes mellitus) and mental health-related variables (perceived status of health, perceived level of stress, subjective cognitive decline, and depression). CI, confidence interval; OR, odds ratio; PSQI, Pittsburgh Sleep Quality Index.

## Data Availability

The dataset used and analyzed in the current study is available from the corresponding author upon reasonable request. Raw data are available on the Korea Community Health Survey website (https://chs.kdca.go.kr/chs/index.do) (accessed on 15 July 2021).
